# 

**Published:** 1902-10

**Authors:** 


### Society Notes.

### West Texas Medical Association.

The twenty-seventh annual meeting will be held on the morning, afternoon and evening of Thursday, October 30th, at San Antonio. Evening session will be followed by a banquet.

### The Southwestern Tri-State Medical Association.

This association will meet in Dallas October 7th and 8th, inst., during the progress of the Texas State Fair. A large attendance is expected. No program received to date. Dr. J. O. McReynolds, Secretary, Dallas, Texas.

### Tri-State Medical Society of Alabama, Georgia and Tennessee.

The fourteenth annual meeting of this popular organization will be held in Birmingham, Ala., Tuesday, Wednesday and Thursday, October 7, 8 and 9, 1902. All are invited to attend. Reduced rates on the certificate plan have been granted by the Southeastern Passenger Association from all points in its territory (south of the Ohio and east of the Mississippi rivers).

### American Medical Association.

New Orleans, La., September 4, 1902.

*To the Editor of the Texas Medical Journal.*

Dear Doctor: As you know, the next meeting of the American Medical Association will take place in New Orleans, May 5-8, 1903.

Dr. Frank Billings, of Chicago, the president, has appointed me the chairman of the Committee on Arrangements. It is my desire to make the New Orleans meeting the best the A. M. A. has ever had. To this end the profession of New Orleans proposes doing its utmost. I feel, however, that it is a matter of both interest and pride to the whole South that this meeting should be a great success. It is with this object in view that I am thus early addressing myself to you as a co-worker in the interests of the medical profession.

The local journal proposes, editorially and otherwise, to stimulate the proper enthusiasm in all whom it may reach. Will you not take the matter up, *con amore,* in the same way, so as to promote a desire among the profession in your section and among your subscribers to attend the meeting. By making it a matter of editorial notice from month to month I believe that the right sort of interest will be excited.

We expect, additionally, to increase the local membership in the A. M. A. with the idea of having a large Southern delegation next May, and we should like your help in this also. If the medical journals of the South persistently work for the meeting we ought to show our Northern guests a spirit of personal interest of which both they and ourselves may be proud.

Fraternally yours,

ISADORE DYER, M. D.

Per A. E.

---

### West Texas Medical Association.

### The Brazos Valley Medical Association.

Easterly, Texas, September 15, 1902.

*Dear Doctor:*

The next meeting of the Brazos Valley Medical Association will be held on the second Tuesday and Wednesday in November, 1902, at Rockdale, Texas.

You are very earnestly requested to prepare a paper on some theme of your own selection, and send title of subject chosen at once to the secretary, Dr. W. B. Briggs, at Easterly, Texas, that the programs may be completed as early as possible. If you doubt your ability to attend the meeting, please send your paper to the secretary by the first of November, that it may be read before the association. Please do not fail to write a paper for this meeting.

W. B. Briggs,
Secretary B. V. M. A.

